# Biosurfactant production by *Myrciaguianensis*endophytic fungi

**DOI:** 10.1186/1753-6561-8-S4-P213

**Published:** 2014-10-01

**Authors:** Messe Elmer Torres da Silva, Crislene Carvalho Nascimento, Sergio Duvoisin Junior, Patrícia Melchionna Albuquerque

**Affiliations:** 1 College of Health Sciences, University of Amazonas, Av. Carvalho Leal 1777, Cachoeirinha, 69065-001, Manaus, AM, Brazil; 2 College of Technology, University of Amazonas, Av. Darcy Vargas 1200, Parque 10, 69065-020 Manaus, AM, Brazil

## 

Surfactants are amphipathic molecules constituted by hydrophobic and hydrophilic regions that usually get distributed on interfaces between fluid phases of different polarities (water/oil and oil/water) [1]. Several compounds with tensoactive properties are synthetized by living organisms (animals, plants and microorganisms), being considered natural surfactants or biosurfactants, which reduce superficial tension and present a significant emulsifying capacity. Endophytic fungi are microorganisms associated to plants, which inhabit, at least during a certain period of time, the interior of vegetal tissues without causing any damage or producing external structures [2]. Plants present a great potential for the obtainment of biologically active compounds. However, there are only a few reports regarding endophytic microorganisms isolated from tropical plants. Among the interesting host species, the genus *Myrcia *has brought the scientific interest due to the presence of a considerable amount of biologically active compounds for the production of antibiotics and antioxidants [3]. Therefore, this work aimed to verify the production of biosurfactants by endophytic fungi isolated from roots and stems of *Myrcia guianensis*. The endophytes were isolated previously [4] and maintained on BDA tubes, being activated on this media at 28°C during 5 to 10 days. It was produced a spore suspension (1,0 × 10^8 ^spores/mL), which was inoculated in Erlenmeyer flasks containing the liquid media - MgSO_4 _(0.5 g/L), Na_2_HPO_4 _(3.0 g/L), KH_2_PO_4 _(1.0 g/L) and yeast extract (1.3 g/L). After autoclaving the media, it was added 0.5 g/L of soybean oil in order to induce the biosurfactant production [5]. The fungi were cultivated in duplicate during 7 days in a shaker at 28°C and 170 rpm. After the experiment, the cultivated media was filtered and the supernatant was used in a quantitative test to determine its emulsifying capacity (E_24_). Eight endophytic fungi were evaluated for the production of biosurfactants (MgC 3.1.1, MgC 3.3.2, MgRe 1.3.3, MgRe 2.3.1, MgRe 2.1.1, MgRe 1.3.1, and MgC 2.1.2). The results showed that four culture media formed emulsions with kerosene, indicating the production of tensoactive molecules by the isolated fungi. The emulsifying index obtained was 64,93% for MgRe 2.3.1, 69,52% for MgC 3.3.2, 70,0% for MgC 3.1.1 and 75,75% for MgRe 1.3.1. It is worthy to mention that the synthetic surfactant sodium dodecyl sulfate, used as positive control, presented a emulsifying index of 78,45% at a 1% solution. Hence, it is possible to conclude that the endophytic fungi isolated from *Myrcia guianensis *were able to produce biosurfactants and that they present a great potential as a source of new products.
